# Key messages of the WHO European Regional Obesity Report

**DOI:** 10.1093/eurpub/ckac129.354

**Published:** 2022-10-25

**Authors:** K Wickramasinghek, J Williams, I Rakovac, G Grosso, M Heinen

**Affiliations:** 1 WHO European Office for NCD, WHO Regional Office for Europe, Copenhagen, Denmark; 2 Department of Biomedical and Biotechnological Sciences, University of Catania, Catania, Italy

## Abstract

Obesity is a complex multifactorial disease defined by excessive adiposity and is linked to an increased risk for many noncommunicable diseases (NCDs). Overweight and obesity affect almost 60% of adults and nearly one in three children in the WHO European Region. Recent estimates suggest that overweight and obesity is the fourth most common risk factor for NCDs in the Region, after high blood pressure, dietary risks and tobacco. It is also the leading risk factor for disability and obesity is linked to greater morbidity and mortality from COVID-19. And obesity is considered a cause of at least 13 different types of cancer including cancers of the breast, colorectum, kidney, liver and ovary, multiple myeloma and meningioma. None of the countries in the Region are on track to achieve the obesity related target set in 2013. Early studies from a number of countries in the Region indicate that the prevalence of overweight and obesity and/or mean body mass index has increased in children and adolescents during the COVID-19 pandemic. This latest WHO European report on obesity examines the growing challenge and impact of obesity in the Region, building on past publications and aligning with initiatives to tackle cancer. The report focuses on managing obesity throughout the life course and tackling obesogenic environments; it also considers more recent challenges, including problematic digital marketing to children and the impact of the COVID-19 pandemic on obesity prevalence. Policy options to prevent obesity are outlined for consideration by Member States together with a suite of population-level approaches. The report highlights the importance of including prevention and control of obesity within measures to build back better in the wake of the COVID-19 pandemic. Whilst highlighting that single intervention will not be sufficient in any country, it examines the challenges faced by countries to implement known interventions to tackle obesity.

